# A preliminary study of the diagnostic efficacy and safety of the novel boring biopsy for brain lesions

**DOI:** 10.1038/s41598-022-08366-y

**Published:** 2022-03-14

**Authors:** Toshihiro Ogiwara, Junpei Nitta, Yu Fujii, Gen Watanabe, Haruki Kuwabara, Masahiro Agata, Hideki Kobayashi, Yoshinari Miyaoka, Satoshi Kitamura, Yoshiki Hanaoka, Tetsuya Goto, Mai Iwaya, Kazuhiro Hongo, Tetsuyoshi Horiuchi

**Affiliations:** 1 Department of Neurosurgery, Shinshu University School of Medicine, 3-1-1 Asahi, Matsumoto, 390-8621 Japan; 2 Department of Neurosurgery, Kobayashi Neurosurgical Hospital, 1-5-21 Miwa, Nagano, 380-0803 Japan; 3 Department of Neurosurgery, St. Marianna University School of Medicine, 2-16-1 Miyamaeku, Kawasaki, 216-8511 Japan; 4 Department of Laboratory Medicine, Shinshu University Hospital, 3-1-1 Asahi, Matsumoto, 390-8621 Japan

**Keywords:** Molecular biology, Neuroscience, Neurology, Oncology

## Abstract

Existing methods for biopsy of intraparenchymal brain lesions, including stereotactic biopsy and open block biopsy, have advantages and disadvantages. We propose a novel biopsy method, called “boring biopsy,” which aims to overcome the drawbacks of each conventional method. This method is less invasive and allows obtaining continuous specimens of sufficient volume. We aimed to assess the feasibility and efficacy of using boring biopsy for intraparenchymal brain lesions. We included 26 consecutive patients who underwent boring biopsy for intraparenchymal lesions. Columnar continuous specimens from the surface of the normal brain tissue to the tumor margin and the center of the lesion were obtained using the boring biopsy method. We used a catheter introducer with original modifications to create a cylindrical biopsy tool for surgery. Columnar continuous specimens were successfully obtained. Histopathological diagnosis was based on cellular changes and differentiation from normal tissues to the core of the lesion and established in all cases. No permanent deficits, major adverse outcomes, or deaths were observed. This novel technique may improve diagnostic accuracy and reduce invasiveness associated with brain biopsy. This method may become the next standard procedure, particularly in some cases where histological evaluation is paramount, and conventional biopsy methods are not suitable.

## Introduction

Advances in radiological imaging of the brain have improved the diagnostic yield in neurosurgery. However, a histopathological confirmation is essential for the definitive diagnosis of brain lesions such as tumors. Surgical intervention is required to remove the lesion and obtain tissue for histopathological evaluation; therefore, it is incumbent upon the neurosurgeon to perform a biopsy that is safe and minimally invasive and involves proper handling of specimens for molecular profiling^1–3^. Although stereotactic needle biopsy is relatively less invasive, the diagnostic accuracy of small biopsy samples is questionable due to the histological heterogeneity of tumors^4^. Surgical biopsy, which involves a craniotomy, is associated with high diagnostic accuracy; however, it is an invasive procedure that carries a high risk of complications. Recently, the usefulness of endoscopic biopsy, which involves neuroendoscopy, has been described^5^. These conventional methods have some advantages and disadvantages in sampling accuracy, approach to deep lesions, sample volume, and invasiveness; the choice of optimal technique depends on context.

The present study evaluated the utility of a new biopsy technique for intraparenchymal brain lesions at overcoming the drawbacks of each conventional method; this novel method is called “boring biopsy” and involves creating a hole by using a revolving tool. The boring biopsy may allow a sufficient length of the specimen to be obtained in continuity for improving diagnostic accuracy and reducing invasiveness associated with conventional methods. Hence, this study aimed to assess the feasibility and efficacy of this novel technique for intraparenchymal brain lesions.

## Results

The clinical characteristics of 26 patients with intraparenchymal brain lesions who underwent boring biopsy are shown in Table 1.

**Table 1:** Clinical characteristics of 26 patients with intraparenchymal brain lesions who underwent boring biopsy.

Case	Age	Sex	Laterality	Location	Maximum tumor diameter (mm)	Length of boring biopsy (mm)	Target sampling	Diagnosis on histopathology	Complications
1	60	F	Left	Temporal	35	27	Success	DA	None
2	18	M	Right	Frontal	27	20	Success	DA	None
3	42	M	Left	Temporal	34	25	Success	DA	None
4	79	M	Left	Occipital	52	25	Success	GBM	None
5	70	F	Right	Parietal	49	37	Success	MS	None
6	35	F	Right	Temporal	20	17	Success	Normal brain	None
7	57	M	Right	Occipital	45	33	Success	DA	None
8	76	M	Right	Frontal	48	24	Success	GBM	None
9	34	F	Right	Frontal	33	31	Success	MS	None
10	75	M	Left	Frontal	38	53	Success	GBM	None
11	29	F	Right	Insula	54	30	Success	DA	None
12	49	F	Left	Temporal	37	20	Success	PCNSL	None
13	85	F	Right	Temporal	72	37	Success	GBM	None
14	66	F	Left	Temporal	57	40	Success	GBM	None
15	39	M	Left	Frontal	63	44	Success	PCNSL	None
16	83	M	Left	Parietal	49	43	Success	Hemorrhagic infarction	None
17	36	F	Left	Frontal	35	20	Success	AA	None
18	55	M	Left	Temporal	62	36	Success	GBM	None
19	77	M	Left	Frontal	84	50	Success	PCNSL	None
20	72	M	Left	Occipital	20	37	Success	PCNSL	None
21	67	M	Right	Frontal	33	27	Success	OD	None
22	68	M	Right	Parietal	41	29	Success	AA	None
23	76	M	Right	Corpus callosum	44	39	Success	GBM	None
24	81	M	Right	Frontal	78	44	Success	AA	Hematoma
25	37	M	Left	Frontal	54	33	Success	DA	None
26	76	M	Left	Thalamus	37	38	Success	PCNSL	Transient hemiparesis

*F* female; *M* male; *DA* diffuse astrocytoma; *GBM* glioblastoma multiforme; *MS* multiple sclerosis; *PCNSL* primary central nervous system lymphoma; *AA* anaplastic astrocytoma; *OD* oligodendroglioma.

The study population included 17 male and 9 female patients in the age range of 18–85 (mean age, 59.3) years at presentation. Accurate, adequate, and sufficient specimens were obtained in all cases. The mean maximum tumor diameter was 46.2 mm (range: 20–84 mm). The mean depth from the brain surface to the deepest biopsy point was 33.0 mm (range: 17–53 mm). A definitive diagnosis based on histopathological assessment was possible in all patients (100%). The histopathological diagnoses included glioma (n = 17), primary central nervous system lymphoma (n = 5), multiple sclerosis (n = 2), and hemorrhagic infarction (n = 1). In one patient, a lesion suspected to be a tumor based on preoperative magnetic resonance imaging findings was histologically confirmed as normal tissue. The location of the lesion was the frontal, temporal, parietal, and occipital lobes, and basal ganglia in 10, 7, 3, 3, and 2 patients, respectively, and the corpus callosum in 1 patient. The targets of the biopsy were the right and left sides in 12 and 14 cases, respectively. Postoperative complications were observed in two (8.3%) patients and included asymptomatic postoperative bleeding on a computed tomography scan in one patient and transient aggravation of hemiparesis in the other. No major adverse events were observed.

## Discussion

Stereotactic needle biopsy is commonly used for intraparenchymal tumors. This method is minimally invasive; neither a craniotomy nor general anesthesia is required. The risk of complications such as brain damage due to retraction and corticotomy is low^6^. In this method, a stereotactic frame is used to guide a needle into the abnormal lesion; biopsy fragments can be obtained from the correct target with precise stereotactic planning.

However, some previous studies suggested that stereotactic needle biopsy is associated with poor diagnostic yield due to sampling error and a small amount of tissue obtained^1,4^. Furthermore, specimens obtained from target points may lead to inaccurate diagnosis because of the histological heterogeneity of the tumor. Although robotic-assisted stereotactic biopsy has been recently shown as accurate, the safety and efficacy profile of this approach in brain neoplasms remains unclear^6^. In addition, the risk of postoperative intracerebral hematomas is a significant disadvantage associated with stereotactic biopsy^6,7^; intracerebral hematomas were reported to be located within the tumor biopsy sampling sites and along the needle trajectory in 36.4% of cases^6^. This complication is likely due to the difficulties associated with preoperative determination of the presence of vessels along the needle trajectory; in addition, hematoma cannot be identified intraoperatively, which the more number of procedure of needle biopsy, surgical risk increases. Difficulties in the visualization of the intraparenchymal structures such as a tumor during stereotactic needle biopsy are among its disadvantages. On the other hand, in recent years, when surgical technique and instruments has advanced, surgical outcomes of stereotactic needle biopsy have also improved. Diagnostic yields were very high (96% in the series of Riche et al. in 1500 patients, 99% in the series of Hamisch et al. in 511 patients)^8,9^. The other tools and techniques which allow diagnostic yield improvement such as, intraoperative histological examination and intraoperative use of 5-Aminolevulinic-Acid-Induced tissue fluorescence have also been reported^10,11^. In addition, the rate of symptomatic complications was low (3%) in the series of Riche et al. and in the literature in general^12^. Our data showed a lower complication equivalent to that in stereotactic biopsy, which has been considered as the safest and least invasive procedures among the conventional methods.

An open biopsy involves obtaining a chunk of block specimen via craniotomy; it is the most common form of brain biopsy. Craniotomy is performed above the abnormal lesion, and the specimens are obtained using an operating microscope. The advantages of this approach include a high diagnostic yield and relatively large sample that can be collected from any site of the lesion. Furthermore, most neurosurgeons are familiar with microsurgery. Open biopsy does not require equipment such as a stereotactic brain surgery system and endoscopes. However, this technique is invasive and can be disorientating due to mechanical deviation and brain shift in deeper and smaller lesions. Open biopsy involves a long skin incision, craniotomy, and a large extent of brain retraction and incision; it is a long procedure performed under general anesthesia.

Endoscopic neurosurgery encompasses endoscopic endonasal approach to skull base lesions and intraventricular surgery (including third ventriculostomy and intracerebral hematoma evacuation in cylinder surgery)^13,14^. Recently, navigation-guided endoscopic biopsy has been used to overcome the drawbacks of needle and open biopsies^7^. Ishikawa et al. reported that endoscopic biopsy is safe and feasible for diagnostic tissue sampling^5^. Navigation-guided endoscopic biopsy is less invasive than open biopsy; the required incision length and operation time are both shorter in the former than in the latter procedure and the risk of brain swelling due to damage to the cortical vein by the bone edge is also reduced^7^. Moreover, a navigation-guided endoscopic biopsy may provide a relatively large sample volume within a relatively short operation time^7^. Recent studies have examined the impact of endoscopic biopsy and endoscopic total resection of brain tumors^15^. The biggest advantage of this method is that the target lesion and surrounding vessels can be visualized using an endoscope. Operators can resect a larger lesion volume without increasing the risk of bleeding; if bleeding occurs, it can be controlled with monopolar or biopsy forceps^7^. Furthermore, a combination of multi-modal techniques, including intraoperative pathological diagnosis and photodynamic diagnosis, can help ensure accurate sampling from small targets^7,15^.

Endoscopic biopsy requires specialist equipment and technique. This technique can obtain point specimens, but not line or plane specimens, and it does not account for lesion heterogeneity, which is also the case in stereotactic biopsy. Endoscopic biopsy can be used with both shallow and deep lesions; however, it is not recommended for tumors mainly occupying the pial surface, which is highly vascular, and where microscopic visualization is superior to endoscopic visualization^7^.

We believe that the key characteristics of the ideal biopsy procedure include (1) accurate sampling, (2) less invasiveness, (3) large continuous line specimens, and (4) visualization of the intraparenchymal structures during surgery^5^. Presently, no surgical method satisfies all these criteria. In a boring biopsy, accurate sampling is achieved by using a navigation system. Furthermore, collecting line instead of point specimens may help obtain a wider range of pathological information, particularly, in cases of highly heterogeneous tumors. The potential implications for the tumor heterogeneity are also resolved with advantage of this larger and cylinder tumor samples. Such continuous specimens cannot be collected by stereotactic biopsy, and in this respect, this unique boring biopsy method is superior to conventional stereotactic biopsy. The boring biopsy is less invasive; it does not require a large skin incision/craniotomy or brain retraction, and its trajectory can be visualized using an endoscope. The present study suggests that this method can be performed by general neurosurgeons without additional training in a specialist technique. Boring biopsy may become the next standard procedure, particularly, in cases that require accurate histological evaluation.

In 2016, the World Health Organization (WHO) formulated an integrated molecular and histological diagnostic framework for many central nervous system neoplasms, which was a major departure from the previous classification system^1,16^. The updated molecular classification has direct implications for prognosis, treatment, and clinical trial eligibility^1^. Accurate biopsy findings are paramount in the era of molecular brain tumor treatment. Neurosurgeons are required to offer safe, less invasive, and cost-effective solutions to obtain tissue samples suitable for molecular analysis. Although several conventional biopsy methods exist, the present method may advance brain tumor treatment, and the results of this study may help expand the indication for boring biopsy in the future. Furthermore, the findings of boring biopsy may have a significant impact on the future classification guidelines for central nervous system neoplasms by WHO.

Meanwhile, molecular diagnostics is likely to play a major role in the upcoming WHO classification to be released in 2021. And in the current era, next generation sequencing panels allow to obtain histomolecular diagnosis according to the WHO classification regardless of the heterogeneity of tumors^17^. Consequently, the heterogeneity of a brain tumor may no longer be an important criterion in large biopsy covering a certain length (including normal tissue). However, other insights may be glimpsed from this informations, such as the composition of the immediate microenvironment of a tumor, e.g. type of cells or altered gene expression that could interact with the tumor cells.

This study was conducted at a single-center and relied upon the expertise of the practicing surgeons. Direct comparison with a control group that used different biopsy techniques was not performed. Further studies are required to validate this approach to biopsy. These studies should include large samples, a careful choice of control groups and procedures, and assessments with computed tomography scans; various disease subtypes and postoperative care plans should be considered^1^. The present evidence should be considered preliminary; further research is required to confirm the clinical relevance of collecting continuous columnar samples. In addition, dedicated tools must be developed for this approach to be accepted as a routine clinical practice. Hence, we are currently collaborating with medical engineers to create such a tool.

However, extending sampling to normal tissue may be not without concerns, even if ethical approval is obtained. In glioma surgery, when deep tumor is resected, superficial normal tissue is usually removed to create a surgical corridor to provide access to a tumor in a non-eloquent area of the brain just like boring biopsy. So, we think this concern is acceptable.

In summary, herein, we presented a novel biopsy technique (“boring biopsy”) for examining intraparenchymal brain lesions. This method is safe and constitutes a feasible approach to histopathological diagnosis; in addition, this method can help achieve high diagnostic accuracy while being less invasive. Over time, this method may become the new standard for biopsy, alongside conventional biopsy methods, specifically, in cases where histological evaluation is paramount; nevertheless, additional multi-center, randomized controlled trials of the various biopsy techniques as well as large scale meta-analyses are required.

## Methods

**Figure 1:**
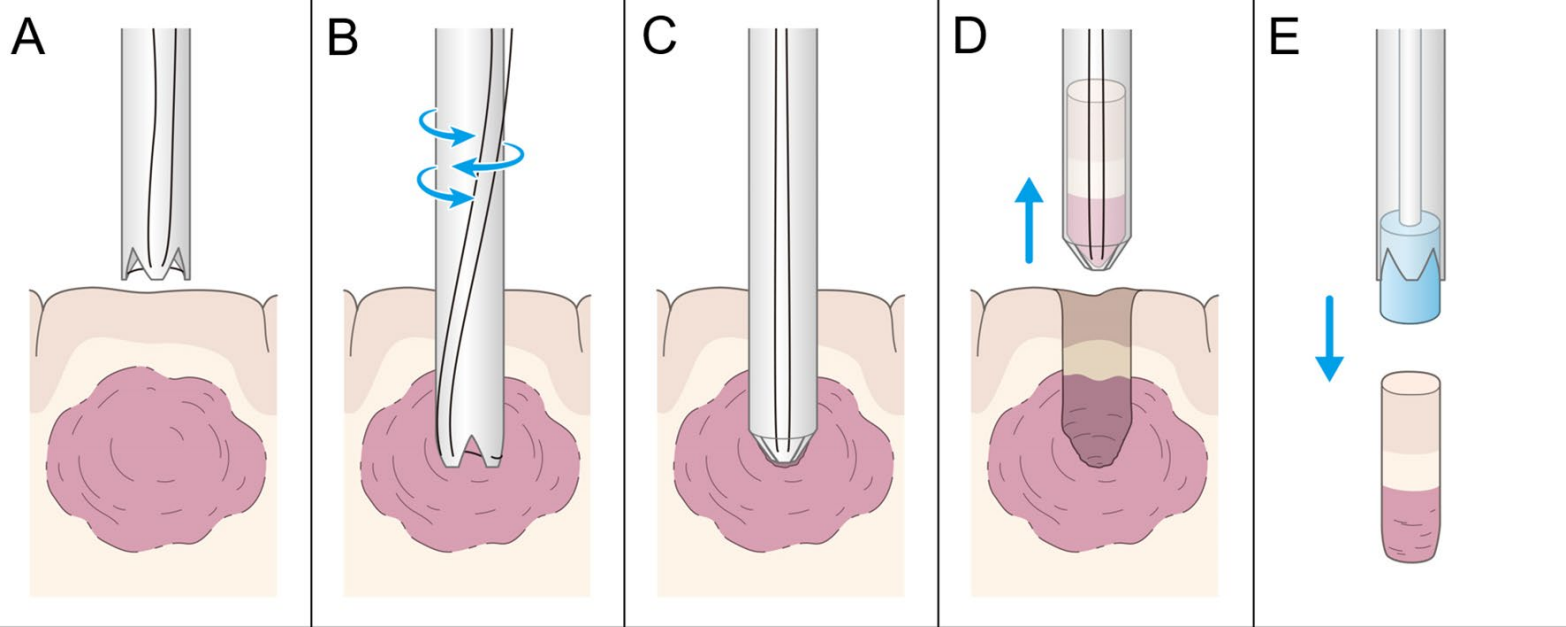
Scheme of boring biopsy method for intraparenchymal lesion, including tool insertion (**A**,**B**), specimen isolation and transection with a closing tip tool (**C**), cylindrical tissue sampling (**D**), and specimen removal (**E**). This method facilitates obtaining a vertically long column of the specimen, ranging from the normal cortex to the core of the lesion.

The boring biopsy is a less invasive method of collecting histological specimens of desirable length, encompassing the superficial areas of the normal brain as well as the tumor margin and center (Fig. 1).

**Figure 2:**
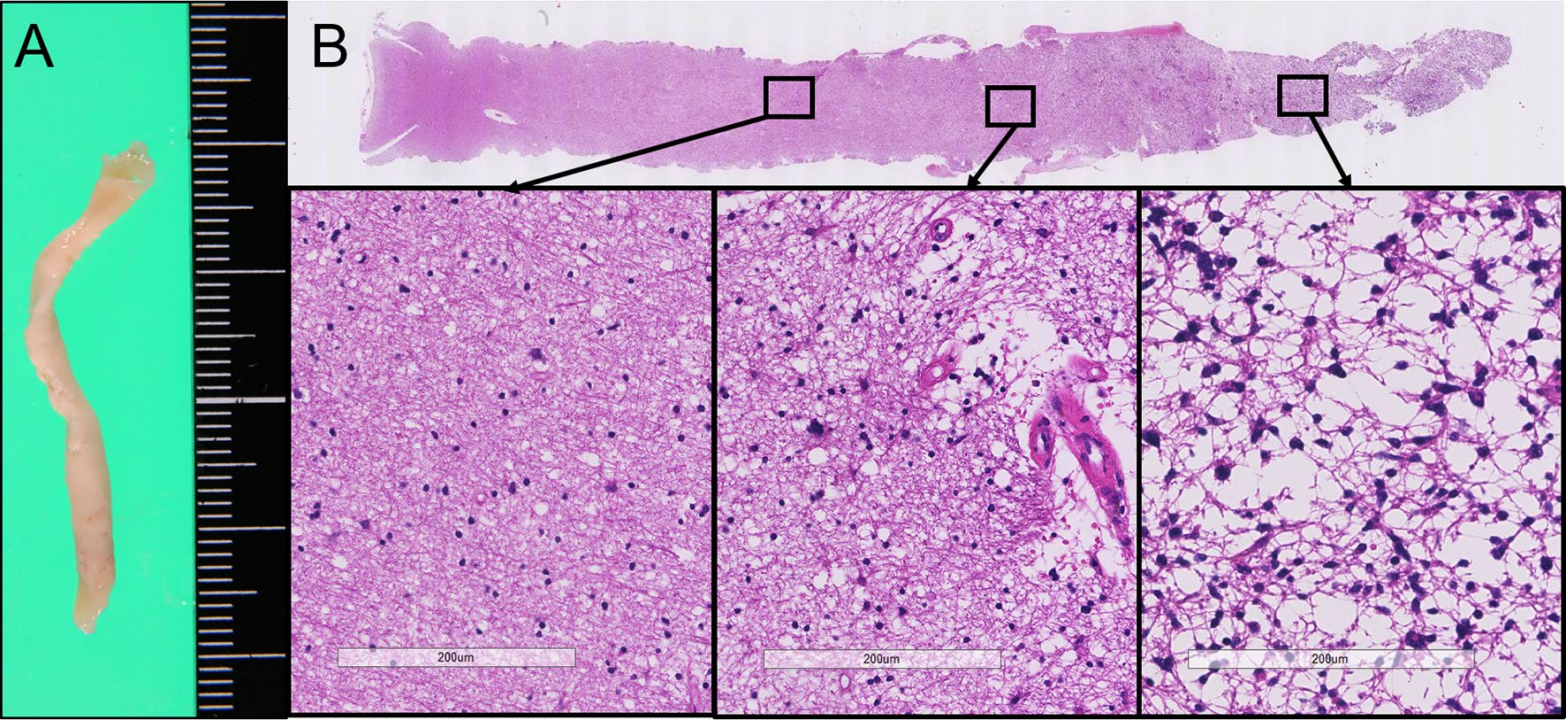
Boring biopsy enables the sampling of a continuous columnar specimen from the surface of the normal brain to the tumor margin and the center of the tumor (measurement marks are defined in millimeters.) (**A**) Cellular changes and differentiation from normal tissues to the center of the tumor. From the border of the tumor to the center of the tumor, the World Health Organization grade rises from 2 and 3 to 4. Hematoxylin & eosin staining showing dense cellularity, nuclear pleomorphism, and microvascular proliferation at the center of the tumor (**B**).

This approach allows for a comprehensive examination of cellular changes and differentiation in normal tissues and across the tumor. Moreover, this approach is essential for a definitive diagnosis and helps in the collection of information on lesion properties and ascertaining the degree of progression (Fig. 2).

**Figure 3:**
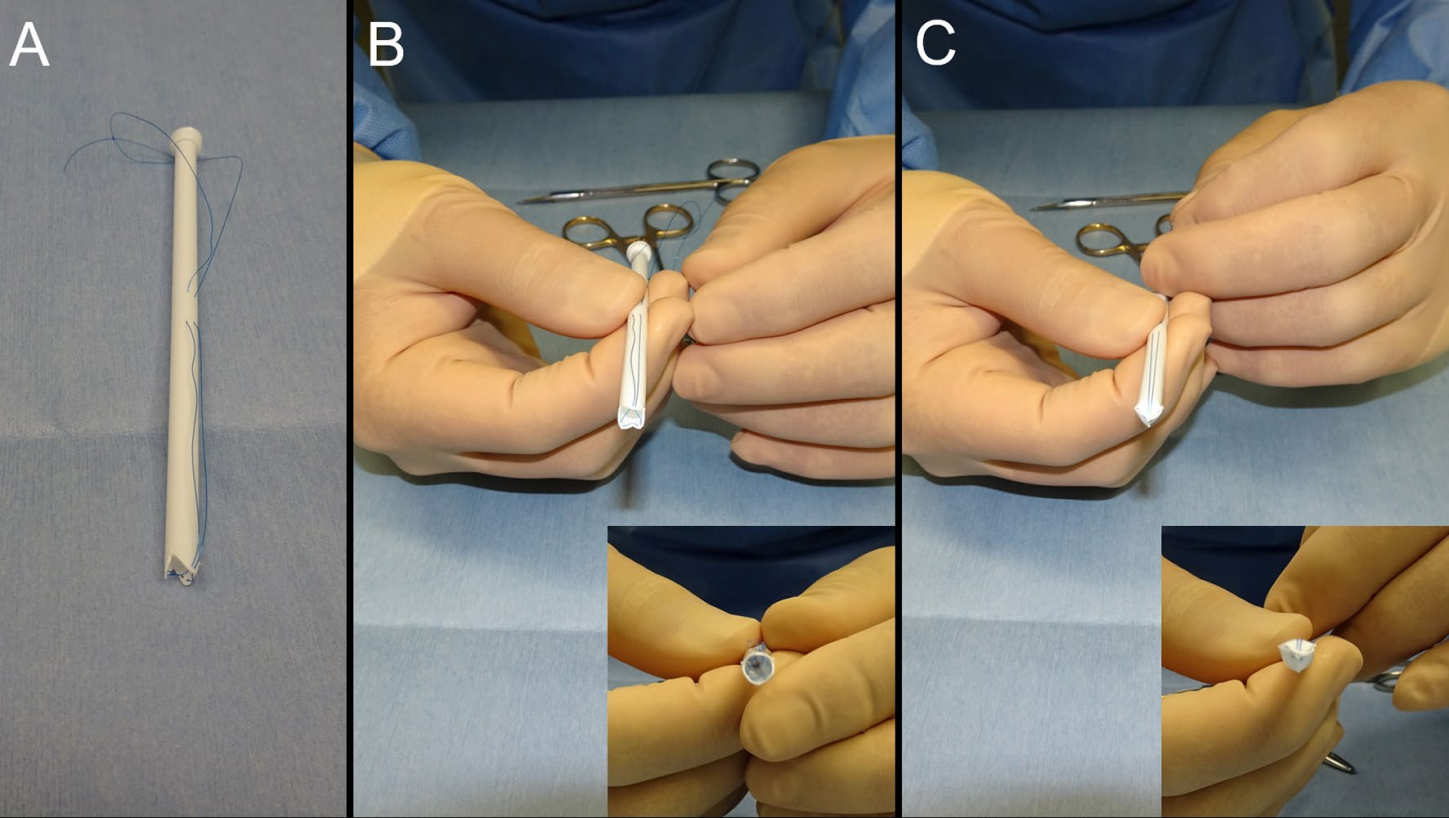
Modified angiographic catheter introducer: a cylindrical biopsy tool (**A**). Open (**B**) and close (**C**) position of the tool tip (achieved through a thread).

This information may help better understand disease pathology and optimize treatment decisions over time. However, a tool suitable for the complete boring biopsy is yet to be developed. Herein, we used an angiographic catheter introducer (11 or 12 French gauge, Medikit CO., LTD., Tokyo, Japan) with modifications to create a cylindrical biopsy tool suitable for surgery (Fig. 3).

### Patient population

Of 62 patients who underwent biopsy for intraparenchymal brain lesions between January 2014 and June 2019 at Shinshu University Hospital and Kobayashi Neurosurgical Hospital, we included 26 consecutive patients who underwent boring biopsy in this prospective study.

Surgical strategy with biopsy for brain lesion was decided at a team conference that included neurosurgeons, neuroradiologists, neuropathologists, and anesthesiologists. Patients were eligible for boring biopsy preferentially if they had intraparenchymal lesions and sufficient tumor resection was not feasible, leaving them without a histopathological assessment. Tumor location was an eligibility criterion. Biopsy for superficial lesions was performed with block biopsy via craniotomy (n = 23). Deep lesions present in the eloquent areas including the brainstem were also biopsied using the stereotactic technique (n = 3). Patients with tumor hypervascularity and/or hard tumor (e.g., severely calcified tumor), as indicated by preoperative radiological findings, were not eligible for boring biopsy, and these patients underwent open block biopsy or endoscopic biopsy (n = 10).

The patient- or tumor-related characteristics of interest were sex, age, tumor location, the accuracy of target sampling, histopathology findings, and complications. The accurate biopsy point was defined as the correspondence between the preoperative target point and resected point, based on postoperative magnetic resonance imaging or computed tomography findings. Ethical approval for systematically extending sampling to normal tissues using the novel tool was obtained from the Ethics Committee of the Shinshu University School of Medicine (Permission number: 3543). All procedures were performed in accordance with the ethical standards of the institutional and/or national research committees and with the 1964 Helsinki Declaration and its later amendments or comparable ethical standards. Informed consent was obtained from all participants.

### Clinical application of boring biopsy (Video 1)

Boring biopsy was performed under general anesthesia through a burr hole or small craniotomy by neurosurgeons certified by the Japan Neurosurgical Society. The procedure involved four steps. First, a preoperative target was identified, and trajectory planning was performed using a neuronavigation system^18^. Findings from preoperative T1-weighted magnetic resonance imaging scan with gadolinium-based contrast medium determined the target lesion. The ideal biopsy trajectory was determined by a neurosurgeon using the iPlan Stereotaxy planning software (version 3.0, BrainLAB AG, Feldkirchen, Germany), aiming to reach the tumor and to avoid any critical structures, such as cerebral sulci, intracranial vessels, and eloquent brain areas. When a navigation system was not available, this step was omitted, and the ideal biopsy trajectory was decided with the guidance of intraoperative ultrasound.

**Figure 4:**
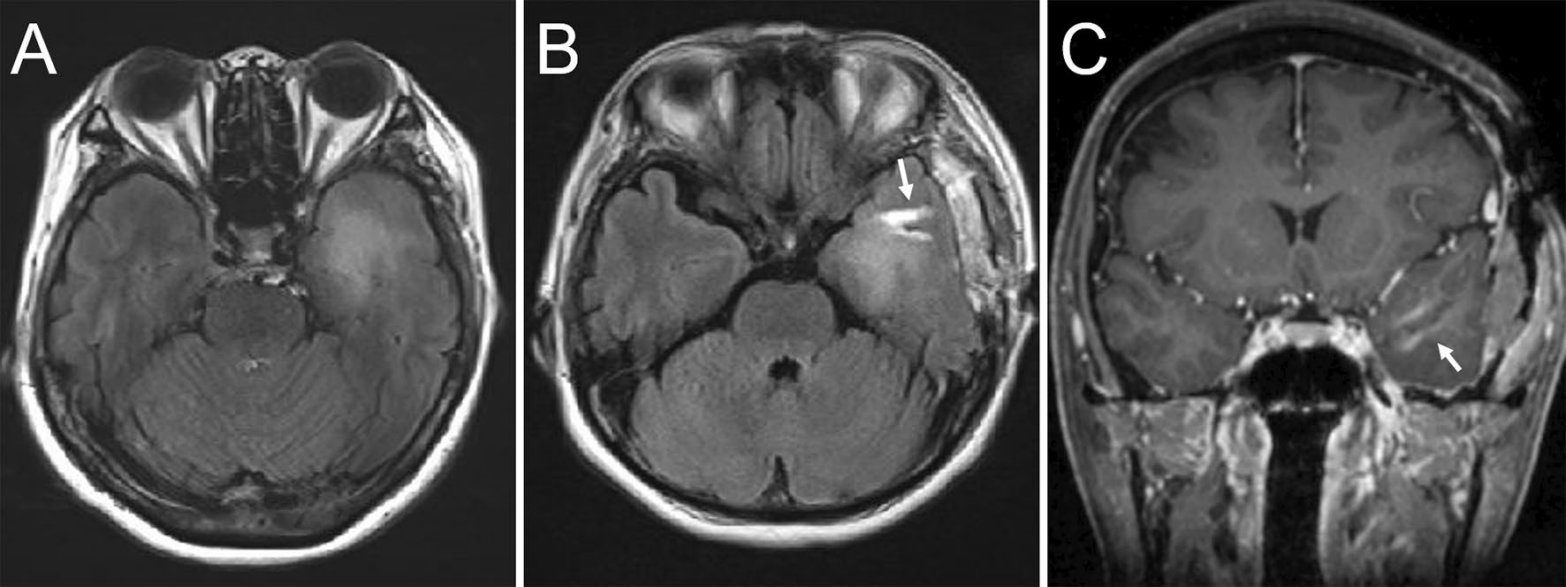
Preoperative magnetic resonance imaging (MRI) scans showing fluid-attenuated inversion recovery hyperintensity lesion at the left temporal lobe (**A**). Postoperative MRI scans, following boring biopsy twice revealed that column cavity from the surface to the core of the lesion is present without hematoma and surrounding brain edema (**B**,**C**).

Second, the boring biopsy tool was handmade intraoperatively. We modified a catheter introducer, which is usually used for angiography, to remove column specimens with a tip closure system (Fig. 3). Tools were modified in a clean surgical field. The tip of the introducer was split in three places; a 5–0 polypropylene monofilament tarn was advanced through the introducer and a stitch was placed approximately 1 cm distal to the insertion depth. Third, we inserted the boring biopsy tool made in the second step. Corticotomy was performed, and the custom tool was inserted toward the lesion via the preplanned trajectory with the aid of navigation guidance. During this procedure, the preplanned trajectory was overlaid on the microscopic surgical field, and the microscope angle was changed until the entry and target points of the trajectory overlapped. A boring biopsy tool was inserted accurately as planned under microscopic view. Fourth, a targeted tissue biopsy fragment was obtained. After identifying the position and direction of the lesion with the navigation system or ultrasound, the boring biopsy tool was inserted manually. Once the target depth was achieved, the tip of the tool was closed to isolate and transect the tissue; finally, the tool was retrieved. Whole these procedures took about 1 h. The removal cavity was inspected with a rigid endoscope, as required. This method helped obtain a vertically long column specimen, reaching from the normal cortex to the core of the lesion (Figs. 1, 4). This specimen contained a continuous normal tumor margin and the target tissue (Fig. 2).

## Supplementary Information

Supplementary Legends.

Supplementary Video 1.
