# Supplementary material for: A preliminary study of the diagnostic efficacy and safety of the novel boring biopsy for brain lesions
Source: Sci Rep. 2022 Mar 14;12:4387. doi: 10.1038/s41598-022-08366-y (PMC8921193; doi:10.1038/s41598-022-08366-y)
Supplement: Supplementary file 1 — Supplementary Legends. [file 41598_2022_8366_MOESM1_ESM.docx]

**Video Legend**

**Video 1**. Clinical application of boring biopsy.
